# Impact of Pelvic Radiotherapy on Gut Microbiota of Gynecological Cancer Patients Revealed by Massive Pyrosequencing

**DOI:** 10.1371/journal.pone.0082659

**Published:** 2013-12-18

**Authors:** Young-Do Nam, Hak Jae Kim, Jae-Gu Seo, Seung Wan Kang, Jin-Woo Bae

**Affiliations:** 1 Department of Life and Nanopharmaceutical Sciences and Department of Biology, Kyung Hee University, Seoul, Republic of Korea; 2 Fermentation and Functionality Research Group, Korea Food Research Institute, Sungnam, Republic of Korea; 3 Department of Radiation Oncology, Seoul National University Hospital, Seoul, Republic of Korea; 4 Cell Biotech Co., Ltd., Seoul, Republic of Korea; 5 Colleges of Nursing, Seoul National University, Seoul, Republic of Korea; Charité, Campus Benjamin Franklin, Germany

## Abstract

Although pelvic irradiation is effective for the treatment of various cancer types, many patients who receive radiotherapy experience serious complications. Gut microbial dysbiosis was hypothesized to be related to the occurrence of radiation-induced complications in cancer patients. Given the lack of clinical or experimental data on the impact of radiation on gut microbiota, a prospective observational study of gut microbiota was performed in gynecological cancer patients receiving pelvic radiotherapy. In the current study, the overall composition and alteration of gut microbiota in cancer patients receiving radiation were investigated by 454 pyrosequencing. Gut microbial composition showed significant differences (*P* < 0.001) between cancer patients and healthy individuals. The numbers of species-level taxa were severely reduced after radiotherapy (*P* < 0.045), and the abundance of each community largely changed. In particular, the phyla Firmicutes and Fusobacterium were significantly decreased by 10% and increased by 3% after radiation therapy, respectively. In addition, overall gut microbial composition was gradually remolded after the full treatment course of pelvic radiotherapy. In this set of cancer patients, dysbiosis of the gut microbiota was linked to health status, and the gut microbiota was influenced by pelvic radiotherapy. Although further studies are needed to elucidate the relationship between dysbiosis and complications induced by pelvic radiotherapy, the current study may offer insights into the treatment of cancer patients suffering from complications after radiation therapy.

## Introduction

More than 50% of cancer patients receive irradiation for cancer treatment [1]. Pelvic irradiation has long been used as a curative or palliative therapy and has been proven to be successful for the treatment of various types of cancer, including abdominal and cervical cancers [2,3]. However, side-effects are common for irradiated patients because irradiation may injure normal tissues of the pelvic skin, distal large bowel, loops of the small intestine, and the urogenital area along with the targeted tumor cells [4]. During and after the radiotherapy period, many patients (i.e., 75% of visceral pelvic cancer patients) suffer symptoms, including diarrhea, mucus discharge, rectal bleeding, tenesmus, and fecal incontinence. These complications may increase the healthcare costs and mortality of cancer patients, with longer hospitalizations and slower cancer treatments [2]. 

While radiation enteropathy is a serious complication, therapeutic strategies are limited because the mechanisms of radiation enteropathy are not well understood. Recent studies aimed to elucidate human-microbiome interactions provided insight into potential therapeutics. Crawford and Gordon revealed the importance of gut microbiota in the occurrence of radiation injury [5]. They showed that germ-free mice were resistant to lethal radiation injury and had less radiation-induced epithelial cell damage as compared to conventional mice with commensal gut microbial flora. The overgrowth of gram-negative bacilli was shown to be essential in the pathogenesis of radiation enteropathy [6]. Johnson et al. reported that bowel irradiation may lead to a general decrease of gut microbiota, imbalance of the gut bacterial community structure, and subsequent pathogenic effects on the epithelial mucosa [7]. However, despite increasing evidence of the relationship between gut microbiota and radiation enteropathy, no comprehensive molecular analyses have been performed to investigate the influence of irradiation on gut microbiota in human cancer patients.

Recent advances in sequencing technology, such as the 454 pyrosequencing approach, provide a faster and simpler way for analyzing microbial communities compared to any other culture-dependent or -independent methods [8-11]. They have been successfully applied to characterize the microbial diversity in various regions of the human body, including the skin [12], oral cavity [13], vagina [14], and intestinal tract [15,16]. Up to now, however, there has been no comprehensive study of the effect of radiotherapy on gut microbiota in cancer patients using this high-throughput technology. Therefore, in the current study, a detailed and comparative analysis of the gut microbial communities of radiation-treated cancer patients was performed. Fecal samples were periodically collected from nine gynecological cancer patients before, during, and after pelvic radiotherapy. These samples were analyzed by 454 pyrosequencing with sample-specific barcoded primers targeting the hyper-variable regions V1/V2 of the bacterial 16S rRNA genes. In addition, the overall shape of the gut microbiota profile of cancer patients was compared to that of healthy individuals. To our knowledge, this is the first molecular ecological investigation elucidating the influence of radiation on the gut microbiota of gynecological cancer patients using a deep sequencing approach. The results of this study will broaden our knowledge about the functions of the host-microbe interaction in radiation injury and will provide insight into both disease pathophysiology and potential therapeutics for cancer patients.

## Materials and Methods

### Sampling and DNA extraction

Fecal samples were provided by nine gynecologic cancer patients (age: 35–63 years) who were undergoing pelvic radiotherapy (Table S1). Only patients not receiving antibiotics, steroids, and immune-suppressants were included in this study. Radiotherapy was delivered at doses of 50.4 Gy per day, five times a week during a 5 week period. Written informed consent was obtained from all participants. The study protocol was approved by the Institutional Review Board of Seoul National University (IRB number: H-1002-059-310). Four sequential stool samples were collected from each patient: before starting treatment (baseline sample, T0), after the first radiotherapy (first radiotherapy sample, T1), at the end of the fifth radiotherapy (last radiotherapy sample, T2), and after radiotherapy (follow-up sample, T3). All the T0 samples were collected in one week before radiotherapy and T3 samples were collected between one month and three months after final radiotherapy. T3 sample of “H” patient was not collected because of taking probiotics after full series of radiotherapy. Each participant collected approximately 5 g of stool into a sterile plastic container and immediately stored the container in a freezer until they brought it to the experimental laboratory. Samples were stored at the laboratory at −80 °C until further processing. The fecal DNAs were extracted using the QIAamp Sool Mini kit (Qiagen, Valencia, CA, USA) and used as the template for PCR amplification.

### Pyrosequencing of bacterial 16S rRNA fragments

To amplify the V1/V2 16S rRNA gene regions [17], a 30ng of purified DNA was amplified with a TOPsimple^TM^ DryMIX solution (Enzynomics, Daejeon, Korea) were amplified with the primer pair 8F (5’-AGAGTTTGAT CCTGGCTCAG-3’) and 338R (5’-TGCTGCCTCC CGTAGGAGT-3’) containing eight base sample-specific barcode sequences (Table S2) and common linker (TC for forward and CA for reverse primer) sequences at the 5’ end [18]. This approach allowed the analysis of PCR products from multiple samples in parallel on a single 454 picotiter plate, and the ability to re-sort the sequences into order [19]. Thermocycling was conducted in a C 1000 Thermal Cycler (Bio-Rad, Hercules, CA, USA) under the following conditions: initial denaturation at 94 °C for 2 minutes; 18 cycles of denaturation at 94 °C for 30 seconds, annealing at 55 °C for 30 seconds, extension at 72 °C for 1 minute, and a final extension at 72 °C for 10 minutes. 

After the PCR reaction, the quality of the amplified products was confirmed by electrophoresis and PCR amplicons were purified with the QIAquick PCR Purification kit (Qiagen, Valencia, CA, USA). An equal quantity (100 ng) of each PCR amplicon tagged with the sample-specific barcode sequences was pooled and subsequently amplified by emulsion PCR before sequencing by synthesis with the massively parallel pyrosequencing protocol [20]. Sequencing was performed through a 454 pyrosequencing Genome Sequencer FLX Titanium (Life Sciences, Branford, CT, USA) according to the manufacturer’s instructions by a sequencing provider (Macrogen, Seoul, Korea). 

### Sequence processing

The sequences generated from pyrosequencing were mainly analyzed with the software MOTHUR [21]. Sequences were filtered by removing sequences with more than one ambiguous base call and retaining only sequences that were 300 nt or longer to minimize the effects of poor sequence quality and sequencing errors. Sample-specific sequences were collected according to the barcode sequences tagged to each sample. The sequences obtained in this study were uploaded and made available through the DNA data bank of Japan (DDJB) under the project ID 72883 (accession numbers for samples: DRS001948-DRS001983).

### OTU determination and taxonomic classification

Trimmed sequences from each barcode bin were aligned using Infernal and associated covariance models obtained from the Ribosomal Database Project Group [22]. The aligned sequences based on secondary structural information were further trimmed to encompass the same V1/V2 regions. This process allowed accurate analysis using the same regions, and simultaneously increased the alignment speed. In addition, potential chimeric sequences were detected and removed with the *chimera.slayer* command of MOTHUR. Sequences were realigned with the SILVA-compatible alignment database (http://www.mothur.org/w/images/9/98/Silva.bacteria.zip). For the data normalization, we randomly extracted 1000 sequences from each sample and these normalized sequences were used for the downstream analysis. The OTUs (90% to 100% sequence similarity) were assigned by using the *cluster* command with the furthest neighbor clustering algorithm. The OTUs defined by a 3% distance level were phylogenetically classified with a modified bacterial RDP II reference database containing 164,517 16S rRNA sequences prepared with TaxCollector (http://www.microgator.org). 

### Community comparison analysis

To examine the variation of gut microbiota during radiotherapy and to compare the overall gut microbial community of healthy individuals to that of cancer patients, the OTU information from each sample was transferred into dendrograms with the *tree.shared* command of MOTHUR. Distances between microbial communities from each sample were calculated with the Jaccard and Yue & Clayton θ coefficients. They were represented by Unweighted Pair Group Method with Arithmetic Mean (UPGMA) clustering trees describing the dissimilarity (1-similarity) between multiple samples. The resulting matrices were also visualized with principal coordinate analysis (PCoA) plots, which indicated what fraction of the total variance in the data was represented by each axis. Variations in the genetic structures of microbial communities between groups (healthy individuals *vs.* cancer patients) and between groups according to radiation therapy stage were analyzed with analysis of molecular variance (AMOVA) to assess significant differences between groups. 

### Calculation of species richness and diversity indices

Shannon’s diversity (H’ = -∑*p*_i_ln(*p*_i_), where *p*_i_ is the proportion of taxon i) [23], ACE, and Chao I richness indices [24], and rarefaction curves [25] were generated with the MOTHUR program. The 3% dissimilarity cut-off value was used for assigning an OTU. Good’s coverage was calculated as G = 1-n/N, where n is the number of singleton phylotypes and N is the total number of sequences in the sample. 

### Reference data

The 16S rRNA gene sequence data of the gut microbial communities of six healthy Korean adult women were downloaded from DDBJ (ftp://ftp.ddbj.nig.ac.jp/ddbj_database/dra/fastq/DRA000/DRA000316) and used in this study as reference data of healthy individuals [15]. 

### Statistical analysis

The significance of observed differences in gut microbial taxa among each group was mainly assessed by a one-way analysis of variance (ANOVA), followed by Student-Newman-Keuls posthoc comparison with GraphPad InStat version 3.05 for Windows (GraphPad Software, San Diego, CA, USA). The results were presented as mean values ± standard errors of the mean (SEMs). Differences were considered to be significant at *P* < 0.05. 

## Results

### Diversity estimation of gut microbiota in gynecological cancer patients

After quality control processes and removing chimeric sequences, we finally obtained 78,650 sequences from this experiment. However, we only used randomly selected 1000 sequences for each sample in the downstream analysis for data normalization.” Table S2 summarizes the number of unique sequences, OTUs, and richness, diversity, and coverage values of each normalized sample. Each individual sample contained an average of 554.3 (standard deviation (SD) = 91.0) unique sequences and 111.7 OTUs (SD = 18.0) at a cut-off level of 97% for the 16S rRNA gene similarity (general bacterial species demarcation). The number of expected OTUs estimated by Chao 1 richness estimator in each sample was considerably higher than the number of observed OTUs (average = 182.0, SD = 34.8), which suggested that additional phylotypes would be identified when all of the existing sequences in each sample were fully inspected. However, when a rarefaction analysis was performed to determine whether all of the OTUs present in the normalized datasets had been sufficiently recovered in the pyrosequencing study, individual rarefaction curves showed a similar pattern of reaching the plateau stage (Figure S1). In addition, Good’s coverage of each individual sample, which was used to estimate the completeness of sampling by a probability calculation based on a randomly selected amplicon sequences, also showed high values (average = 95.7%, SD = 0.8%) with a 97% species-level-phylotype threshold 

### Differences of gut microbiota between gynecological cancer patients and healthy individuals

**Figure 1:**
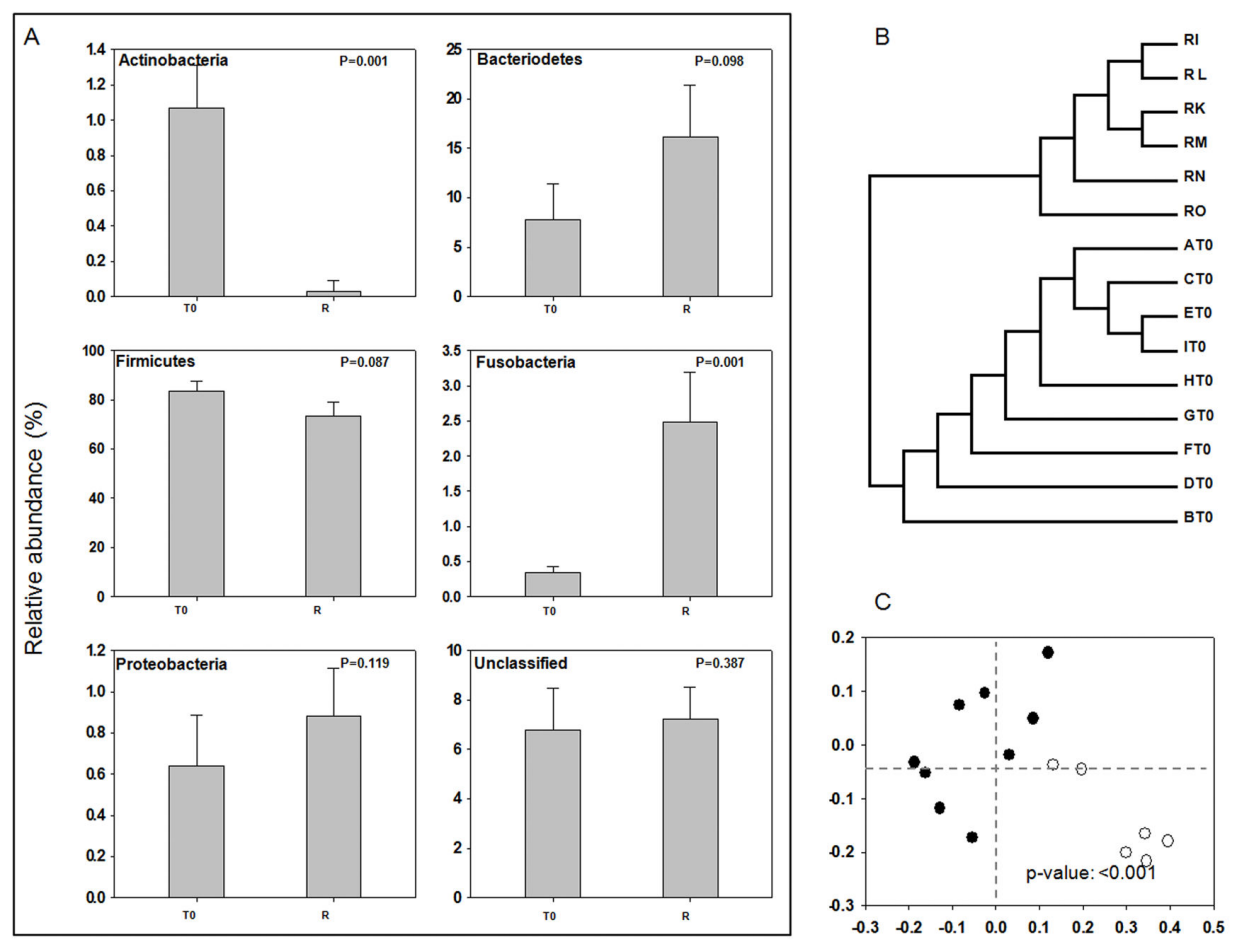
Comparison of bacterial communities between healthy individuals and patients. Relative abundances of six phylum-level taxa are compared (A). Each bar represents the mean value of abundance (± SEM). *P*-values showing the significance of differences between cancer patient and healthy individual groups are shown at the upper part of each graph. Overall species-level bacterial communities were compared and clustered by the UPGMA algorithm (B) and visualized by PCoA plots (C) with the Jaccard coefficient; RI, RK, RL, RM, RN, and RO represent healthy individuals, and AT0 to IT0 represents gynecological cancer patients before radiotherapy.

To compare gut bacterial communities between healthy individuals and gynecological cancer patients, the relative abundance of each phylum-level bacterial taxon in fecal samples collected from nine gynecological cancer patients (T0) and data from six healthy women retrieved from our previous study [15] were investigated (Figure S2A). Cancer patients and healthy individuals were associated with nine bacterial phyla, Actinobacteria, Bacteroidetes, Firmicutes, Fusobacteria, Lentisphaerae, Proteobacteria, Synergistetes, Tenericutes and Verrucomicrobia, and unclassified bacteria, which are the most commonly encountered bacterial phyla in the human intestinal tract [12,26]. However, the relative abundances of the dominant phylum differed between the two groups. Actinobacteria in cancer patients was thirty times higher than that in healthy individuals, whereas Bacteroidetes, Fusobacteria, and Proteobacteria in cancer patients were 2.1, 7.4, and 1.4 times lower than those in healthy individuals, respectively. When the relative abundances of each phylum were compared between cancer patients and healthy individuals, Actinobacteria (*P* = 0.001) and Fusobacteria (*P* = 0.001) showed significant differences between the two groups (Figure 1A). 

The gut bacterial communities were largely populated by 15 bacterial phylogenetic families with an average abundance of 74.4% (Max = 84.1%, Min = 66.2%) in cancer patients and 83.2% (Max = 89.1%, Min = 75.8%) in healthy individuals (Figure S2B). Analysis by ANOVA revealed significantly different results for the richness of bacterial family groups between cancer patients and healthy individuals (Table 1). Among the 15 tested family groups, Prevotellaceae, Clostridiaceae, Eubacteriaceae, Oscillospiraceae, Fusobacteriaceae, Enterococcaceae, Streptococcaceae were significantly different between cancer patients and healthy controls (*P* < 0.05). The relative abundances of the families Clostridiaceae (2.5 times) and Eubacteriaceae (4.8 times) were significantly higher, whereas Prevotellaceae (2.9 times) and families Oscillospiraceae (3.0 times) and Fusobacteriaceae (6.3 times) were significantly lower in cancer patients compared to healthy individuals. 

**Table 1:** Family-level differences between gynecologic cancer patients and healthy controls.

**Family level taxon**	**Abundance in Patients (%)**	**Abundance in Healthy (%)**	**P-value**
Ruminococcaceae	42.5 ± 4.0	36.4 ± 7.4	0.136
Prevotellaceae	4.1 ± 3.7	11.7 ± 4.6	0.026
Lachnospiraceae	4.4 ± 0.8	7.7 ± 2.4	0.076
Veillonellaceae	2.3 ± 0.7	9.3 ± 5.9	0.085
Clostridiaceae	6.0 ± 1.7	2.4 ± 0.5	0.018
Bacteroidaceae	2.7 ± 0.4	3.1 ± 1.6	0.144
Eubacteriaceae	3.9 ± 1.4	0.8 ± 0.3	0.025
Lactobacillales bacterium	0.1 ± 0.0	3.4 ± 1.6	0.001
Oscillospiraceae	0.8 ± 0.5	2.4 ± 0.8	0.033
Erysipelotrichaceae	2.3 ± 1.8	0.5 ± 0.2	0.164
Fusobacteriaceae	0.3 ± 0.1	1.9 ± 0.4	0.007
Porphyromonadaceae	0.6 ± 0.2	1.1 ± 0.4	0.107
Butyrate-producing bacterium	2.1 ± 0.6	2.6 ± 1.3	0.327
Enterococcaceae	1.1 ± 0.4	0.0 ± 0.0	0.028
Streptococcaceae	1.0 ± 0.5	0.1 ± 0.0	0.007

We next compared the overall composition of the gut microbial community of cancer patients with that of healthy individuals. UPGMA dendrograms and ordination plots (PCoA) describing the similarity of the samples to each other were generated with the representative 16S rRNA gene sequences corresponding to species-level OTUs of the T0 samples in this study and those of the six previously analyzed healthy individuals. Figure 1B shows the UPGMA tree representing the similarity of bacterial membership of cancer patients and healthy individuals. Remarkably, cancer patients and healthy individuals clustered separately from each other. The PCoA plot also showed a clear separation between cancer patients and healthy individuals (Figure 1C). An AMOVA test was performed to determine whether the centers of the plots representing a group were more separated than variation among samples of the same group [27]. The results indicated that the microbial communities of cancer patients and healthy individuals showed significant differences (*P* < 0.001). 

### Impact of radiation therapy on gut microbiota of gynecologic cancer patients

**Figure 2:**
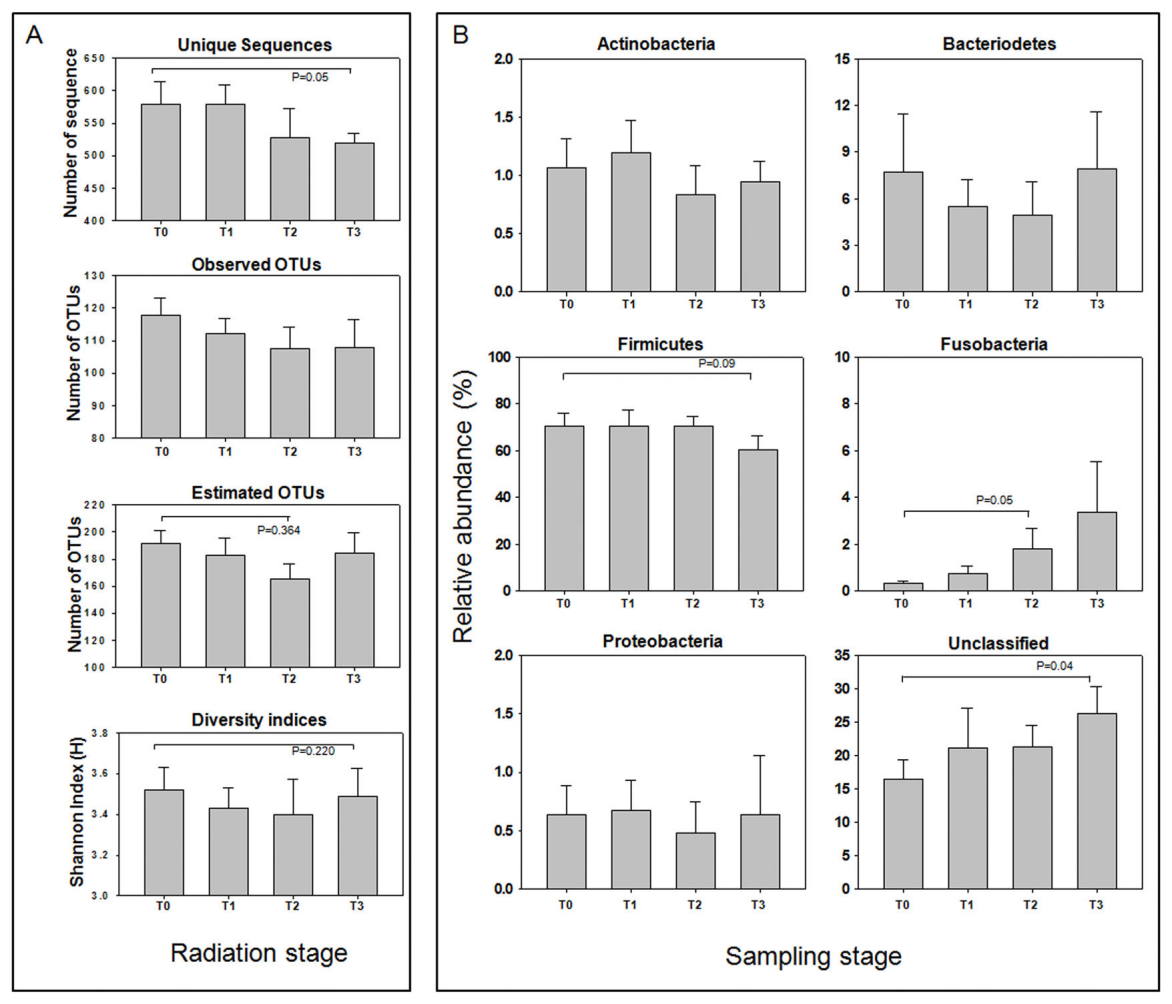
Impact of radiation therapy on the gut microbial community. Unique sequences, observed OTUs, estimated OTUs, and diversity indices (H) according to radiation therapy were analyzed (A). The mean number (± SEM) of each parameter is shown. *P*-values between T0 and T3 are marked. Changes of six major phylum-level taxa during radiation therapy are represented (B). The mean abundance (± SEM) is shown. *P*-values representing significant differences are only shown for each taxonomic group. T0 = before radiation therapy, T1 = after first radiation therapy, T2 = after fifth radiation therapy, and T3 = follow-up samples.

We examined the impact of radiation therapy on the gut microbial community of gynecological cancer patients. We first investigated the impact of radiation therapy on the richness and diversity of gut microbiota in cancer patients. Figure 2A shows the temporal changes in the unique sequences, observed OTUs, estimated OTUs, and diversity indices (H) during radiation therapy in cancer patients. Compared to initial samples (T0), the number of unique sequences was slightly decreased after the first radiation therapy (T1), dramatically decreased during radiation (T2), and resulted in a 10.4% decrease in follow-up samples (T3). Although the number of observed OTUs markedly differed between samples, it showed a decreasing trend through the radiotherapy period.This decreasing tendency was also identified in the number of estimated OTUs and Shannon diversity index (H). Statistical analyses to verify the significance of these differences revealed that the number of unique sequences were reduced between T0 and T3 (P = 0.06) and estimated OTUs were significantly reduced through the radiotherapy (*P* = 0.04). Therefore, the richness of the gut microbial community in cancer patients may be affected by radiation therapy. In the current study, two individuals were not taken chemo treatment during radiotherapy. Therefore we compare microbial richness between chemo treated and non-treated cancer patients according to the radiotherapy stage. The number of OTUs of chemo treated patients changed from 116.4 (SD=18.0) to 112.1 (SD=15.2) and that of non-treated patients changed from 122.0 (SD=12.7) to 112.5 (SD=14.8) through the 1^st^ radiotherapy. In addition, estimated richness by Chao1 estimator of chemo treated patient changed from 192 (SD=33.2) to 185 (SD=37.5) and that of non treated patients changed from 189 (SD=23.3) to 176 (SD=49.5). While the data were not statistically analyzed because of insufficient number of samples, richness of gut microbiota in non-chemo treated patients were rather reduced during radiotherapy. Therefore, changes of gut microbiota in gynecological cancer patients might seem to be caused by radiotherapy.

We next examined the impact of radiation therapy on the gut microbial community composition in cancer patients. Figure S3 shows the temporal change of all phylum-level taxa, and Figure 2B shows the change in the relative abundances of major phylum-level taxa during radiation therapy. Whereas Actinobacteria and Proteobacteria showed a similar fluctuating pattern, Firmicutes steadily decreased and Fusobacteria and unclassified bacteria gradually increased during radiation therapy, respectively. The relative abundance of phylum Bacteroidetes gradually decreased during radiation therapy but was largely increased at T3. When statistical analysis was performed to confirm whether the differences between the stages were significant, Firmicutes, Fusobacteria and unclassified bacteria showed significant differences. The relative abundance of Fusobacteria at T2 was 6.0 times higher than that at T0 (*P* = 0.05).Unclassified bacteria gradually increased and finally showed 9.9% increment compared to T0 samples (*P* = 0.04). By contrast, Firmicutes was decreased by 10.1% through radiation therapy (*P* = 0.09). 

**Figure 3:**
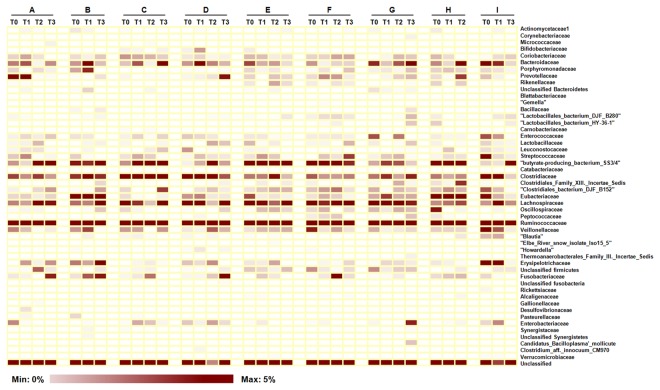
Changes of gut bacterial family level taxa in gynecological cancer patients through the radiotherapy. Each column in the heatmap represents a sample from nine cancer patients. Three or four samples from the same individuals were grouped together in parallel. Each row represents a family level taxon. The color intensity of the panel is proportional to the abundance of certain taxon (max 5%). The family level taxon name is represented on the right side of heatmap.

Figure 3 shows the temporal changes of major family-level taxa during radiation therapy. Although the family-level gut microbial communities of some patients showed large fluctuations, the overall composition of family-level gut microbiota after the first radiation therapy changed little compared to the initial stage. However, the shape of the family-level gut microbial communities gradually changed through the radiotherapy. Table S3 shows the relative abundances of 15 major family-level taxa at each time point and *P*-values representing the degree of differences. Compared to T0, family Eubacteriaceae in each sample at T2, and T3 were significantly decreased (*P* < 0.032). Fusobacteriaceae significantly increased at T2 and Streptococcaceae significantly increased at T1 compared to T0. Family level taxon Veillonellaceae, Enterococcaceae, Lactobacillales bacterium and Butyrate-producing bacterium at T0, T1, and T2 were not different from each other, but the T0 and T3 samples showed significant differences (*P* = 0.050). In addition to inspecting phylum- and family-level changes corresponding to radiation therapy, we used “metastats” to determine whether there were any species-level phylotypes that were differentially represented between the samples from each stage [28]. Table 2 shows the relative abundance of each species-level taxon and *P*-values between the two groups (with each stage compared to baseline). Although there were many significantly different species-level taxa, we have only represented major taxa with relative abundance greater than 0.1% and *P*-values < 0.05. At T1, only eight species-level taxa were affected, and the difference of abundance was less than 0.4%. However, at T2, nine species-level taxa were affected, and the maximum variation between T0 and T2 was 3.5%. In addition, nineteen species-level taxa were significantly changed after the full series of radiation therapy. Through radiotherapy, the average variations of the relative abundance in species-level taxa compared to T0 were 0.21% (T1), 1.06% (T2), and 0.18% (T3). Four species-level taxa were decreased at T1, and 5 and 2 species-level taxa were decreased at T2 and T3, respectively. In addition, 4, 4, and 17 species-level taxa were increased at T1, T2, and T3, respectively. 

**Table 2:** Significantly different OTUs between T0 (before treatment) and T1 (first radiation therapy), between T0 and T2 (fifth radiation therapy), and between T0 and T3 (follow-up) samples (data only represent OTUs with > 0.1% abundance).

**Sample**	**Taxon name**	**Abundance in T0 (%)**	**Tested stage (%)**	**Difference (%)**	**p-value**
T0 vs. T1	*Ruminococcus* sp. CO28	0 ± 0	0.4 ± 0.4	0.4	0.001
	*Roseburia* sp. DJF VR77	0 ± 0	0.3 ± 0.3	0.3	0.001
	*Ruminococcus* sp. CO41	0 ± 0	0.2 ± 0.2	0.2	0.001
	*Lachnospira pectinoschiza*	0 ± 0	0.1 ± 0.1	0.1	0.001
	*Weissella confuse*	0.3 ± 0.2	0 ± 0	0.3	0.001
	*Enterobacter* sp. mcp11b	0.2 ± 0.2	0 ± 0	0.2	0.001
	*Klebsiella pneumonia*	0.1 ± 0.1	0 ± 0	0.1	0.001
	*Adlercreutzia equolifaciens*	0.1 ± 0.1	0 ± 0	0.1	0.001
T0 vs. T2	Butyrate-producing bacterium SS2/1	1.2 ± 0.4	4.1 ± 1.3	2.9	0.009
	*Ruminococcus callidus*	1.0 ± 0.5	0 ± 0	1.0	0.03
	*Dialister* sp. E2 20	1.0 ± 0.4	0 ± 0	1.0	0.013
	Human intestinal firmicute CB47	0.9 ± 0.3	4.4 ± 1.8	3.5	0.025
	*Eubacterium eligens*	0.8 ± 0.4	0.1 ± 0.1	0.7	0.032
	*Eubacterium hallii*	0.1 ± 0.1	0 ± 0	0.1	0.041
	*Actinomyces odontolyticus*	0.1 ± 0.1	0 ± 0	0.1	0.046
	*Lactobacillus murinus*	0.1 ± 0.1	0 ± 0	0.1	0.039
	Clostridiales bacterium DJF CP67	0 ± 0	0.2 ± 0.1	0.2	0.009
T0 vs. T3	*Prevotella stercorea*	0.3 ± 0.3	0 ± 0	0.3	0.001
	*Clostridium* sp. BG-C36	0.1 ± 0.1	0 ± 0	0.1	0.001
	*Ruminococcus* sp. DJF VR52	0 ± 0	0.6 ± 0.3	0.6	0.001
	*Prevotella copri*	0 ± 0	0.3 ± 0.3	0.3	0.001
	*Ruminococcus* sp. CO28	0 ± 0	0.3 ± 0.2	0.3	0.001
	Butyrate-producing bacterium T1-815	0 ± 0	0.2 ± 0.1	0.2	0.001
	*Roseburia inulinivorans*	0 ± 0	0.2 ± 0.1	0.2	0.001
	*Bacteroides* sp. CCUG 39913	0 ± 0	0.2 ± 0.2	0.2	0.001
	Swine fecal bacterium FPC110	0 ± 0	0.2 ± 0.2	0.2	0.001
	*Faecalibacterium* sp. DJF VR20	0 ± 0	0.2 ± 0.2	0.2	0.001
	*Clostridium methylpentosum*	0 ± 0	0.1 ± 0.1	0.1	0.001
	*Oscillospira* sp. BA04013493	0 ± 0	0.1 ± 0.1	0.1	0.001
	*Candidatus Bacilloplasma*	0 ± 0	0.1 ± 0.1	0.1	0.001
	Clostridiales bacterium A2-162	0 ± 0	0.1 ± 0.1	0.1	0.001
	*Coriobacterium* sp. CCUG 33918	0 ± 0	0.1 ± 0.1	0.1	0.001
	*Amphibacillus* sp. YIM-kkny6	0 ± 0	0.1 ± 0.1	0.1	0.001
	Lachnospiraceae bacterium DJF RP14	0 ± 0	0.1 ± 0.1	0.1	0.001
	*Clostridium leptum*	0 ± 0	0.1 ± 0.1	0.1	0.001
	*Ruminococcus* sp. CS1	0 ± 0	0.1 ± 0.1	0.1	0.001

Although radiation therapy obviously impacts the gut microbial community of cancer patients, which specific taxa are altered during radiation therapy remains ambiguous. For example, microorganisms in the genus Ruminococcus were slightly increased at T1 and eliminated at T2 but three Ruminoccocal microorganisms were identified again at T3 In addition, some species-level taxa included in the same genus showed opposite patterns of variation (increment/decrement). For example, *Clostridium* sp. BG-C36 was eliminated, but *C. methylpentosum and C. leptom* increased through irradiation. Therefore, irradiation might not affect specific groups of gut microbiota but might broadly affect the microorganisms, which deviate from the normal healthy status depending on the gut microbial composition of the individual. 

**Figure 4:**
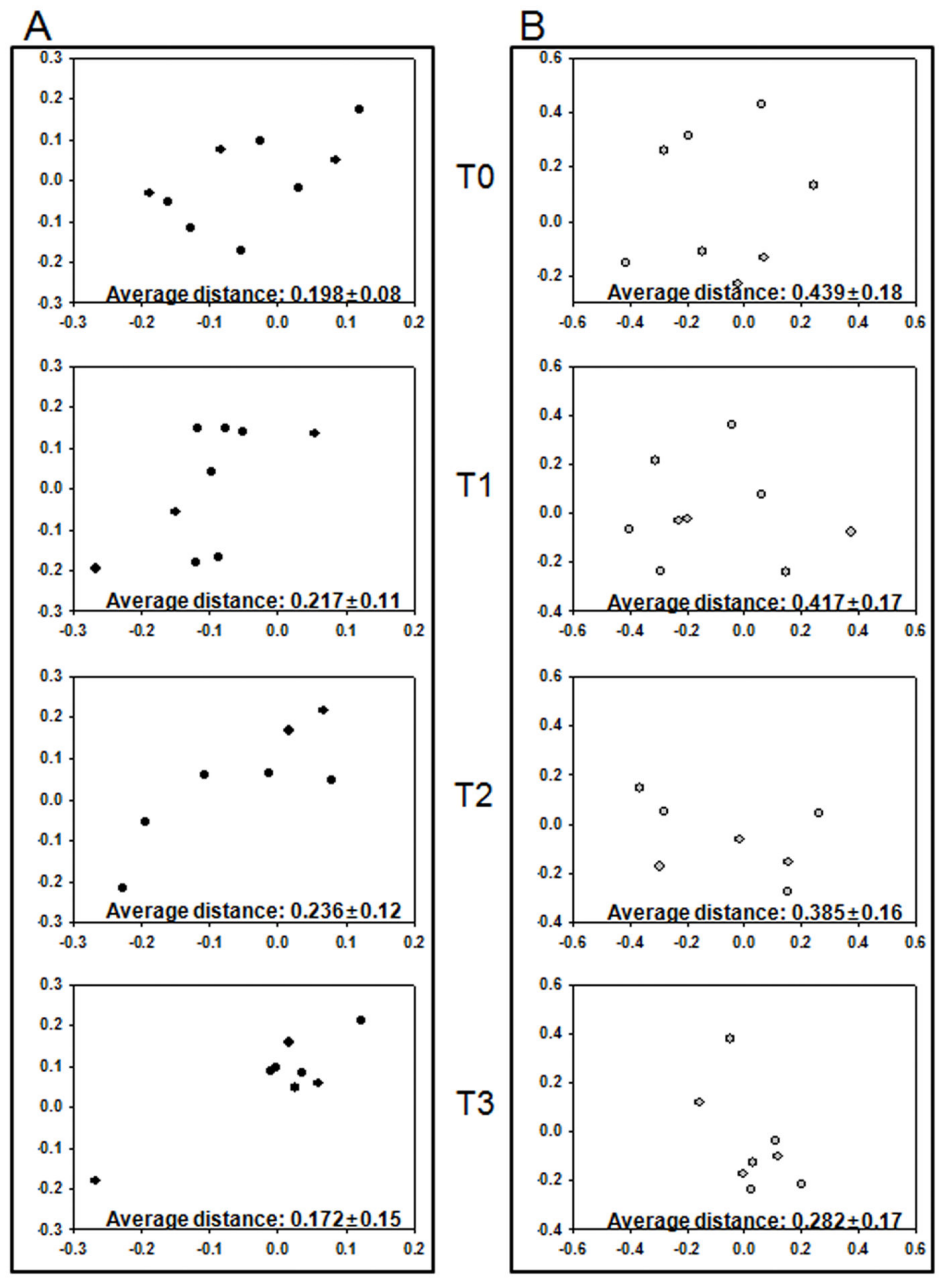
PCoA comparison of gut microbiota during radiation therapy. Distances between all communities were clustered with the Jaccard (A) and thetayc coefficient (B) and visualized with PCoA plots. All plots in a stage (T0, T1, T2, and T3) are separately marked in the different graphs to represent the change of similarity between communities in a stage. Average distances (± SD) between all plots are represented at the lower part of each graph.

Finally, we investigated the patterns of the overall microbial community in cancer patients according to radiation therapy. To compare bacterial communities, distance matrices were calculated from cancer patient samples by using jclass and thetayc coefficients and assessed with PCoA. Figure 4 shows the PCoA plots of each stage of radiation therapy. The microbial community memberships of each individual at each stage were not largely changed through the treatment period (Figure 4A). Analysis by AMOVA revealed that the differences of the gut microbial community at each stage compared to the initial stage were not significant (*P* ≥ 0.322). However, the F ratio, which indicated whether the centers of clouds representing a group were more separated than the variation among samples of the same treatment, increased from 0.796 (T0 *vs.* T1) to 0.992 (T0 *vs.* T2) and 1.038 (T0 *vs.* T3). Although the differences between the initial stage and those of each radiotherapy step were not significant, increments of the F ratio indicated that the memberships of the gut microbiota in cancer patients were gradually changed through the irradiation. 

The PCoA results of the gut microbial community structures of cancer patients during radiation therapy showed larger variations than those of gut microbial community memberships (Figure 4B). However, the F ratios of the structural PCoA comparing the initial stage and each treatment stage were comparatively lower than the membership PCoA. The reason for this result is because the plotting patterns did not undergo a spatial shift toward any of the points far from the PCoA plots of the initial stage but instead converged to a point of the PCoA plots. Therefore, we calculated all of the distances between the PCoA plots in each stage. The average distance was largely reduced during radiation therapy, from average ± SD of 0.198 ± 0.08 at T0 to 0.172 ± 0.15 at T3 in the microbial community membership, and from 0.439 ± 0.18 at T0 to 0.282 ± 0.17 at T3 in the microbial community structure. These results suggested that radiation therapy affected the highly individual-specific gut microbiota of the cancer patients, remolding the shape of the gut microbial community to be more similar among all cancer patients. 

## Discussion

Dysbiosis of the gut microbiota is associated with various diseases, including inflammatory bowel disease (IBD) [29], colon and colorectal cancers [30,31], type 1 diabetes (T1D) [32], type 2 diabetes (T2D) [33], and obesity [34]. The metabolites and antigens produced by gut microbiota play important roles in cancer risk and intestinal inflammation through interactions with host metabolisms and immunity. For example, hydrogen sulfide [35], acetaldehyde [36], and secondary bile acids [37] produced by gut microbiota could affect the progression of colorectal cancer by inciting colonic inflammation and tumor-inducing toxicities. 

Host factors also affect the composition of the gut microbiota. A recent study using MyD88^KO^ NOD mice models showed that knockout mice had a significantly lower Firmicutes/Bacteroidetes ratio and an increased number of bacterial families, including Lactobacillaceae, Rikenellaceae, and Porphyromonadaceae, compared to MyD88-sufficient mice [32]. Paneth cells play important roles in host defense mechanisms against inflammatory and infectious diseases of the host intestinal tracts. Reports have shown that the secretion of antimicrobial peptides, such as defensin, by these cells is regulated by the autonomic nervous system of the host and may be affected by stress [38,39]. Manichanh et al. reported that the gut microbial profiles of control (healthy) individuals were grouped together and separate from those of abdominal cancer patients in a cluster analysis of DGGE banding patterns, suggesting that the gut microbial compositions might be determined by the health status of the individual [40]. 

In the current study, the relative abundances of predominant phyla and family level taxon in fecal samples showed large difference between gynecological cancer patients and healthy individuals. Community comparison with species-level phylotypes also revealed a clear separation of gut microbial memberships and structures between cancer patients and healthy individuals. The report of Manichanh et al., in which abdominal cancer patients and healthy individuals contained different gut microbial features, is somewhat convincing because a change in the health status of the intestinal tract, such as chronic inflammation or abnormal function of the epithelial cells, might directly affect the gut microbial community [40]. However, the significant differences in gut microbiota between gynecological cancer patients and healthy individuals found in the present study were quite interesting because there was no direct causality between gynecological cancer and gut microbiota. Therefore, our finding of different gut microbial community compositions between the two groups implies that changes of health status can affect the overall shape of gut microbiota even when the location of disease development is far from or not related to the intestinal tracts. 

The use of radiation therapy for abdominal and pelvic cancers has frequently been found to increase the risk of radiation enteritis, resulting in longer hospitalization times and obstructing the prompt cure of cancer patients [41]. Previous studies have investigated the influence of irradiation on gut microbiota by assuming that changes in gut microbiota probably affect the developmental course of radiation enteropathy [6,7]. A recent study using germ-free animals showed a linkage between gut microbiota and irradiation-induced enteritis [5]. Although it is known that the NF-kB signaling pathway is able to be activated in response to radiation [42], commensal microorganisms may regulate inflammatory responses. For example, *Bacteroides thetaiotaomicron* and *Bifidobacterium infantis* are able to suppress NF-kB activation, whereas microorganisms in the *Clostridium* XIVa group have been reported to attenuate inflammation of the gut epithelium [43,44]. 

Johnson et al. investigated the impact of radiation on gut microbiota in a mouse model. They reported that the quantities of several bacterial groups were significantly reduced at an early time but gradually recovered after irradiation, even though the total bacterial count was not changed after irradiation [7]. Although they identified quantitative changes of specific groups of gut microbiota after radiation therapy, they did not report which microorganisms were imbalanced by irradiation because they only dealt with specific bacterial groups, such as aerobic, anaerobic enterobactericeae, and *Lactobacillus* groups, with a culture-dependent method. Moreover, although Manichanh et al. reported that the gut microbiota of cancer patients was largely changed after radiotherapy, particularly in patients with diarrhea [40], they did not compare the gut microbial community of healthy individuals and that of cancer patients after a full treatment course of radiation. 

In the current study, we separately compared the gut microbiota of healthy individuals with that of cancer patients before radiotherapy, after the first radiotherapy, after the fifth radiotherapy, and at follow-up. Through the test period, the diarrhea indices of cancer patient increased from 3.7 ± 3.7 (T0) to 59.3 ± 12.2 (T2), and the shapes of gut microbiota in cancer patients largely changed. Interestingly, the shapes of the gut microbiota of cancer patients were gradually remolded. While all most patient suffered diarrhea symptom with dramatic change of gut microbial community after radiotherapy, there was a person who did not have diarrhea symptoms. It is not certain that diarrhea is caused by disruption of the balance between gut microbiota or altered gut motility and enzyme secretion as side-effects of radiotherapy. However, our data suggest that radiation therapy has the potential to re-mold gut microbiota. Therefore, there are needs to identify the relationship between diarrhea symptoms and gut microbial changes during radiotherapy in the follow-up study.

Although irradiation is one of the most promising medical treatments for cancer patients, radiation-induced injuries are common. Therefore, the information presented in this study will be helpful for the treatment of cancer patients receiving irradiation and suffering from radiation-induced injury. The diarrhea values of cancer patients gradually increased according to radiation therapy. However, the gut microbial communities were reconstructed after radiation therapy. Therefore, the results of the current study indirectly support the view that the acute diarrhea caused by radiation therapy is related to reconstruction of the resident gut microbiota. Although our preliminary observations need to be independently confirmed in a larger number of patients, we may be able to prepare successful therapeutic strategies against gynecological cancer if we find proper radiotherapy conditions that do not cause enteritis but still reduce cancer size. 

## Supporting Information

Table S1 **Characteristics of cancer patients.**
(DOC)

Table S2 **Number of unique sequences, observed diversity richness (OTUs), estimated richness (Chao 1 and ACE), diversity index (Shannon’s), and sample coverage (Good’s coverage) for normalized 16S rRNA pyrosequencing data of gynecological cancer patients.**
(DOC)

Table S3 **Changes of relative abundance in major family level taxon during radiation therapy.**
(DOC)

Figure S1 **Rarefaction curves for each sample of gynecologic cancer patients calculated at species level (97% sequence similarity) clustering.**
(TIF)

Figure S2 **Phylum and family level abundance profiles of cancer patient and healthy individuals using 16S rRNA sequence classification.** Columns reflect the percentage of 16S rRNA sequences assigned to each phylum (A) and family level taxon (B) classified by MOTHUR with a modified 16S rRNA database from Ribosomal Database Project (RDP).
(TIF)

Figure S3 **Change of phylum level abundance profiles of nine cancer patients using 16S rRNA sequence classification through the radiation therapy.** Columns reflect the percentage of 16S rRNA sequences assigned to each phylum classified by MOTHUR with a modified 16S rRNA database from Ribosomal Database Project (RDP). T0=before radiation therapy, T1=after 1^st^ radiation therapy, T2=after 5^th^ radiation therapy and T3=follow-up samples.
(TIF)
