# Supplementary material for: Impact of Pelvic Radiotherapy on Gut Microbiota of Gynecological Cancer Patients Revealed by Massive Pyrosequencing
Source: PLoS One. 2013 Dec 18;8(12):e82659. doi: 10.1371/journal.pone.0082659 (PMC3867375; doi:10.1371/journal.pone.0082659)
Supplement: Table S1 — Characteristics of cancer patients. (DOC) [file pone.0082659.s001.doc]

| Patient | Diagnosis | FIGO^*^ stage | Age | RT dose (Gy) | Chemotherapy |
| --- | --- | --- | --- | --- | --- |
| A | Cervical cancer | IB1 | 46 | 50.4 | Fluorouracil + cisplatin |
| B | Cervical cancer | IB1 | 60 | 50.4 | Weekly cisplatin |
| C | Cervical cancer | IB1 | 51 | 50.4 | Paclitaxel + carboplatin |
| D | Cervical cancer | IB1 | 49 | 50.4 | Paclitaxel + carboplatin |
| E | Cervical cancer | IIA | 45 | 50.4 | Etoposide + cisplatin |
| F | Cervical cancer | IIB | 64 | 50.4 | Genexol + carboplatin |
| G | Cervical cancer | IIB | 55 | 50.4 | Genexol + carboplatin |
| H | Endometrial cancer | IA | 56 | 50.4 | Not received |
| I | Endometrial cancer | IB | 50 | 50.4 | Not received |

*FIGO: International Federation of Gynecology and Obstetrics (FIGO) system.
