# Supplementary material for: Impact of Pelvic Radiotherapy on Gut Microbiota of Gynecological Cancer Patients Revealed by Massive Pyrosequencing
Source: PLoS One. 2013 Dec 18;8(12):e82659. doi: 10.1371/journal.pone.0082659 (PMC3867375; doi:10.1371/journal.pone.0082659)
Supplement: Table S2 — Number of unique sequences, observed diversity richness (OTUs), estimated richness (Chao 1 and ACE), diversity index (Shannon’s), and sample coverage (Good’s coverage) for normalized 16S rRNA pyrosequencing data of gynecological cancer patients. (DOC) [file pone.0082659.s002.doc]

| Patient | Sample ID | Barcode | Unique | OTUs | Chao1 | ACE | Shannon | Good's  coverage |
| --- | --- | --- | --- | --- | --- | --- | --- | --- |
| A | AT0 | AACCAAGG | 672 | 113 | 204 (156, 306) | 242 (201, 302) | 3.4 (3.3, 3.5) | 95.2% |
|  | AT1 | AACCATCG | 733 | 118 | 228 (171, 346) | 239 (200, 298) | 2.8 (2.6, 2.9) | 94.7% |
|  | AT2 | AACCGCTA | 451 | 95 | 130 (111, 173) | 146 (122, 194) | 3.2 (3.1, 3.2) | 96.3% |
|  | AT3 | AGACAGTG | 517 | 107 | 201 (151, 309) | 241 (198, 306) | 3.3 (3.2, 3.4) | 95.3% |
| B | BT0 | AAGCGGTA | 568 | 87 | 143 (111, 217) | 132 (109, 177) | 2.8 (2.7, 2.9) | 96.5% |
|  | BT1 | AAGGCGTA | 612 | 97 | 183 (134, 296) | 231 (189, 292) | 3.5 (3.4, 3.6) | 96.0% |
|  | BT3 | CATGCTAG | 534 | 127 | 195 (160, 268) | 243 (207, 296) | 3.9 (3.9, 4.0) | 95.3% |
| C | CT0 | AAGGTTGG | 514 | 99 | 153 (123, 223) | 142 (120, 184) | 3.5 (3.5, 3.6) | 96.4% |
|  | CT1 | AATAGCGG | 457 | 87 | 125 (103, 177) | 162 (134, 206) | 3.2 (3.1, 3.3) | 96.9% |
|  | CT2 | AATAGGCG | 781 | 85 | 138 (108, 205) | 138 (111, 191) | 2.8 (2.7, 2.9) | 96.3% |
|  | CT3 | CCAACGTA | 426 | 87 | 169 (123, 273) | 255 (203, 329) | 3.1 (3.0, 3.1) | 95.9% |
| D | DT0 | ACACAGAG | 760 | 124 | 216 (172, 300) | 335 (277, 414) | 3.3 (3.2, 3.4) | 94.0% |
|  | DT1 | ACACCTGA | 698 | 123 | 184 (153, 247) | 188 (160, 239) | 3.5 (3.4, 3.6) | 95.2% |
|  | DT2 | ACAGACAG | 452 | 88 | 136 (109, 201) | 132 (110, 175) | 2.8 (2.7, 2.9) | 96.6% |
|  | DT3 | CCAAGGAA | 505 | 82 | 109 (92, 151) | 112 (96, 146) | 3.0 (2.9, 3.1) | 97.5% |
| E | ET0 | ACAGCAGA | 523 | 130 | 228 (177, 336) | 236 (202, 285) | 3.8 (3.8, 3.9) | 95.0% |
|  | ET1 | ACAGTCTG | 564 | 126 | 177 (151, 234) | 218 (188, 264) | 3.8 (3.8, 3.9) | 95.6% |
|  | ET2 | ACAGTGAG | 544 | 122 | 197 (158, 278) | 248 (209, 305) | 3.7 (3.6, 3.8) | 95.2% |
|  | ET3 | CCAATACG | 559 | 95 | 138 (113, 198) | 151 (130, 187) | 3.4 (3.3, 3.5) | 96.8% |
| F | FT0 | ACCACATG | 688 | 138 | 185 (161, 235) | 190 (167, 230) | 3.8 (3.7, 3.9) | 95.5% |
|  | FT1 | ACCACTAG | 547 | 125 | 235 (177, 357) | 243 (207, 297) | 3.6 (3.5, 3.7) | 94.9% |
|  | FT2 | ACCAGTTG | 481 | 117 | 192 (152, 277) | 223 (189, 274) | 3.7 (3.6, 3.8) | 95.5% |
|  | FT3 | CCAATTGG | 538 | 116 | 214 (161, 329) | 215 (182, 264) | 3.5 (3.4, 3.6) | 95.4% |
| G | GT0 | ACCATGCA | 534 | 124 | 217 (169, 314) | 259 (218, 318) | 3.6 (3.5, 3.7) | 94.8% |
|  | GT1 | ACCTAGCA | 545 | 109 | 165 (135, 229) | 169 (141, 221) | 3.5 (3.4, 3.6) | 95.9% |
|  | GT2 | ACCTCAAG | 462 | 117 | 173 (143, 240) | 194 (167, 235) | 3.7 (3.6, 3.8) | 95.9% |
|  | GT3 | CCATATGG | 561 | 156 | 231 (193, 309) | 211 (188, 252) | 4.2 (4.2, 4.3) | 94.9% |
| H | HT0 | ACCTCTTG | 467 | 131 | 205 (167, 282) | 190 (164, 236) | 3.8 (3.7, 3.9) | 95.1% |
|  | HT1 | ACCTGATG | 526 | 102 | 141 (119, 193) | 138 (120, 174) | 3.3 (3.2, 3.4) | 96.6% |
|  | HT2 | ACCTGTAG | 527 | 129 | 193 (160, 262) | 227 (194, 275) | 3.9 (3.8, 4.0) | 95.3% |
| I | IT0 | ACGTGTTG | 493 | 113 | 172 (139, 244) | 186 (160, 227) | 3.7 (3.6, 3.8) | 96.1% |
|  | IT1 | ACTGCACA | 536 | 123 | 211 (164, 309) | 217 (186, 263) | 3.7 (3.6, 3.8) | 95.3% |
|  | IT3 | CGAACCTA | 516 | 94 | 217 (145, 393) | 229 (186, 293) | 3.5 (3.4, 3.5) | 96.0% |
