# Supplementary material for: Impact of Pelvic Radiotherapy on Gut Microbiota of Gynecological Cancer Patients Revealed by Massive Pyrosequencing
Source: PLoS One. 2013 Dec 18;8(12):e82659. doi: 10.1371/journal.pone.0082659 (PMC3867375; doi:10.1371/journal.pone.0082659)
Supplement: Table S3 — Changes of relative abundance in major family level taxon during radiation therapy. (DOC) [file pone.0082659.s003.doc]

| Family level taxon | Abundance (%) | | | |  | P-value | | |
| --- | --- | --- | --- | --- | --- | --- | --- | --- |
|  | T0 | T1 | T2 | T3 |  | T0 vs. T1 | T0 vs. T2 | T0 vs. T3 |
| Ruminococcaceae | 42.5 ± 4.0 | 43.5 ± 5.2 | 40 ± 5.4 | 35.1 ± 5.4 |  | 0.302 | 0.268 | 0.084 |
| Prevotellaceae | 4.1 ± 3.7 | 0.9 ± 0.6 | 1.2 ± 0.5 | 1.0 ± 0.5 |  | 0.188 | 0.298 | 0.424 |
| Lachnospiraceae | 4.4 ± 0.8 | 4.6 ± 0.8 | 5.0 ± 1.8 | 7.3 ± 1.7 |  | 0.482 | 0.500 | 0.124 |
| Veillonellaceae | 2.3 ± 0.7 | 1.4 ± 0.4 | 1.1 ± 0.4 | 0.9 ± 0.2 |  | 0.135 | 0.062 | 0.027 |
| Clostridiaceae | 6.0 ± 1.7 | 5.2 ± 1.4 | 8.8 ± 5.1 | 3.0 ± 0.6 |  | 0.245 | 0.340 | 0.105 |
| Bacteroidaceae | 2.7 ± 0.4 | 3.6 ± 1.3 | 2.9 ± 1.5 | 6.4 ± 3.7 |  | 0.430 | 0.150 | 0.138 |
| Eubacteriaceae | 3.9 ± 1.4 | 4.5 ± 2.3 | 1.5 ± 0.9 | 1.7 ± 1.1 |  | 0.365 | 0.032 | 0.019 |
| Lactobacillales bacterium | 0.1 ± 0.0 | 0.1 ± 0.1 | 0.1 ± 0.1 | 0.4 ± 0.2 |  | 0.482 | 0.457 | 0.049 |
| Oscillospiraceae | 0.8 ± 0.5 | 0.4 ± 0.2 | 0.5 ± 0.3 | 0.6 ± 0.2 |  | 0.482 | 0.416 | 0.481 |
| Erysipelotrichaceae | 2.3 ± 1.8 | 1.3 ± 0.6 | 0.8 ± 0.3 | 1.3 ± 0.8 |  | 0.218 | 0.375 | 0.500 |
| Fusobacteriaceae | 0.3 ± 0.1 | 0.8 ± 0.3 | 1.8 ± 0.9 | 3.4 ± 2.2 |  | 0.188 | 0.056 | 0.500 |
| Porphyromonadaceae | 0.6 ± 0.2 | 0.8 ± 0.4 | 0.3 ± 0.1 | 0.4 ± 0.2 |  | 0.329 | 0.111 | 0.220 |
| Butyrate-producing bacterium | 2.1 ± 0.6 | 4.4 ± 1.7 | 5.3 ± 1.6 | 4.6 ± 1.2 |  | 0.201 | 0.077 | 0.050 |
| Enterococcaceae | 1.1 ± 0.4 | 0.6 ± 0.2 | 0.6 ± 0.3 | 0.3 ± 0.1 |  | 0.395 | 0.101 | 0.017 |
| Streptococcaceae | 1.0 ± 0.5 | 1.2 ± 0.2 | 0.7 ± 0.2 | 0.6 ± 0.4 |  | 0.043 | 0.262 | 0.089 |
