# Supplementary figures and images for: Impact of Pelvic Radiotherapy on Gut Microbiota of Gynecological Cancer Patients Revealed by Massive Pyrosequencing
Source: PLoS One. 2013 Dec 18;8(12):e82659. doi: 10.1371/journal.pone.0082659 (PMC3867375; doi:10.1371/journal.pone.0082659)

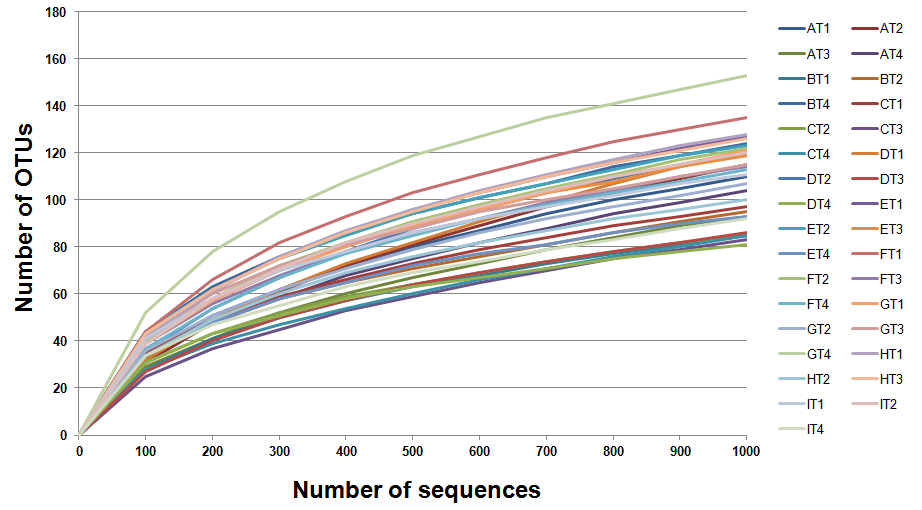

Supplement: Figure S1 — Rarefaction curves for each sample of gynecologic cancer patients calculated at species level (97% sequence similarity) clustering. (TIF) [file pone.0082659.s004.tif]

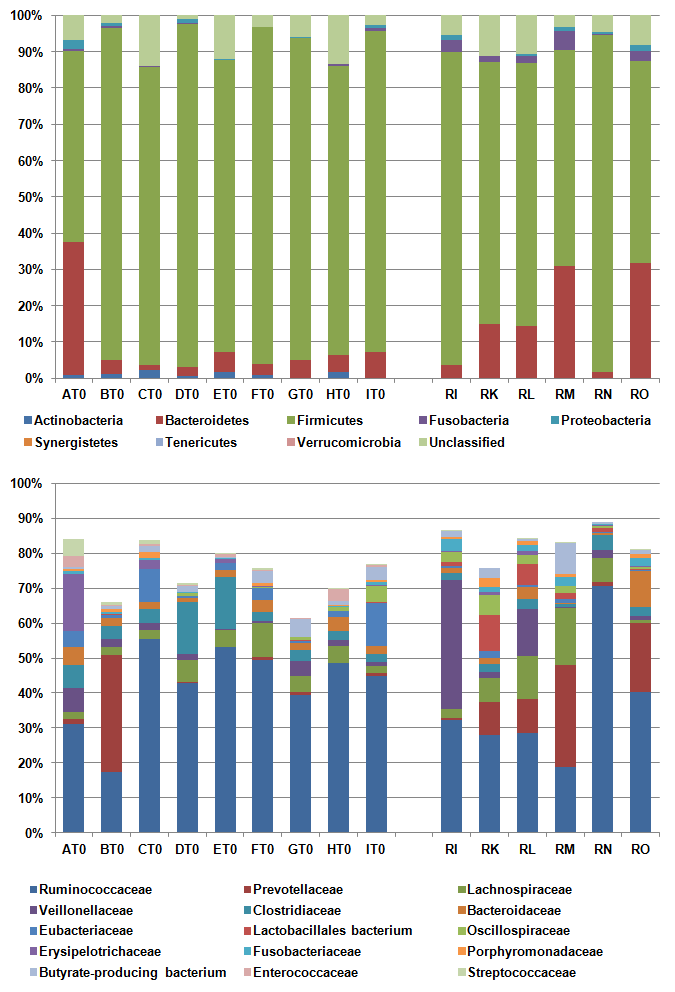

Supplement: Figure S2 — Phylum and family level abundance profiles of cancer patient and healthy individuals using 16S rRNA sequence classification. Columns reflect the percentage of 16S rRNA sequences assigned to each phylum (A) and family level taxon (B) classified by MOTHUR with a modified 16S rRNA database from Ribosomal Database Project (RDP). (TIF) [file pone.0082659.s005.tif]

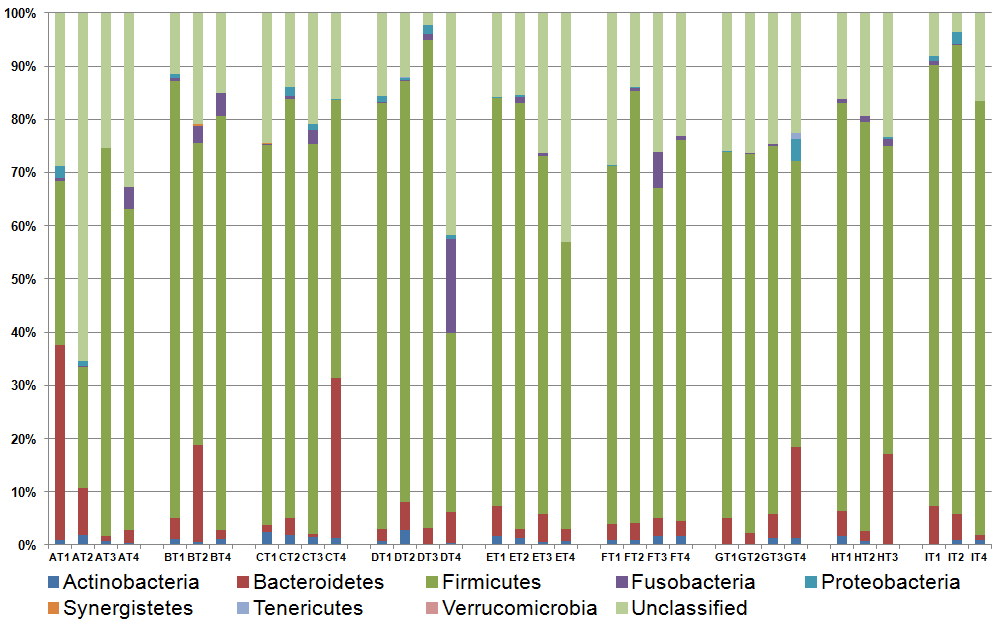

Supplement: Figure S3 — Change of phylum level abundance profiles of nine cancer patients using 16S rRNA sequence classification through the radiation therapy. Columns reflect the percentage of 16S rRNA sequences assigned to each phylum classified by MOTHUR with a modified 16S rRNA database from Ribosomal Database Project (RDP). T0=before radiation therapy, T1=after 1st radiation therapy, T2=after 5th radiation therapy and T3=follow-up samples. (TIF) [file pone.0082659.s006.tif]
